# “I feel *[so alone]* nothing” – emotional vulnerability and detachment as transdiagnostic key characteristics of patients with chronic tinnitus: a schema mode model approach

**DOI:** 10.3389/fpsyt.2024.1375813

**Published:** 2024-06-18

**Authors:** Benjamin Boecking, Petra Brueggemann, Birgit Mazurek

**Affiliations:** Charité – Universitatsmedizin-Berlin - Tinnitus Center, Berlin, Germany

**Keywords:** tinnitus, schema therapy, schema mode model, perceived stress, anxiety, depression, psychological therapy

## Abstract

**Background:**

Gold-standard approaches for chronic tinnitus involve hearing amplification measures and psychological therapy, where applicable. Whilst schema therapy is accumulating evidence as a transdiagnostically useful treatment framework, its applicability for patients with chronic tinnitus has not yet been examined. The present study (a) explores latent dimensions of psychological distress in a sample of chronic tinnitus patients, and (b) examines whether the schema mode model might explain these dimensions – thus constituting a potentially helpful conceptualization and treatment framework.

**Methods:**

*N* = 696 patients with chronic tinnitus completed the Tinnitus Questionnaire, Tinnitus Handicap Inventory, Hospital Anxiety and Depression Scale, Perceived Stress Questionnaire and ICD-10 Symptom Rating. As criterion, patients further completed the Schema Mode Inventory (SMI-r) – which assesses psychological constructs linked to negative self-beliefs (“parent modes”), primary emotions resulting from unmet psychological needs (“child modes”), and secondary emotional or behavioral attempts to reinstate or maintain psychological equilibrium (“coping modes”). A *varimax*-rotated principal axis factor analysis grouped the primary item pool. Factor scale scores were then correlated with the SMI-r.

**Results:**

A three-factor solution explained 37.4% of variance and represented 78% of the included items. Following item content examination, the factors represented (1) General emotional distress, (2) Tinnitus-attributed emotional distress, and (3) Socio-audiological impairment. Factors 1|2 correlated highly (*r* = 0.70), Factors 2|3 moderately (*r* = 0.62). Linked to the schema mode model, Factor 1 correlated highly with the “vulnerable child” (*r* = 0.78), and moderately with the “parent”, “angry child”, and “detached protector” modes (0.53 < *r* < 0.65). Factor 2 correlated moderately with the “vulnerable child” (*r* = 0.53). Factor 3 was largely uncorrelated with SMI-r scores – although a low correlation with the “detached protector” warrants further examination.

**Conclusion:**

“General” and “tinnitus-attributed” emotional distress correlate highly – warranting holistic (not symptom-specific) psychological case conceptualization and treatment planning. Viewed from a schema mode perspective, the “vulnerable child” explains substantial variance across both dimensions. Consequently, autobiographically anchored, unmet emotional needs and emotional detachment constitute key treatment targets. Social-audiological impairment should be multimodally conceptualised and treated with hearing aids and psychological support measures, as applicable.

## Introduction

Chronic tinnitus is a common and frequently disabling symptom which denotes “the conscious awareness of a tonal or composite noise for which there is no identifiable corresponding external acoustic source” [(1) p. 1]. Depending on a person’s internal psychological context, the symptom can be very distressing and place a significant burden on healthcare systems (2).

Often (though not always), chronic tinnitus occurs alongside high frequency hearing loss (3) – and both phenomena can contribute to psychological distress in emotionally vulnerable individuals. According to current treatment guidelines, chronic tinnitus is best treated with a combination of hearing aids and psychotherapy, where indicated (4–6). Evidence for the latter centers on cognitive-behavioral therapies (CBTs) as gold standard treatments (7). CBTs are a group of psychological treatment approaches that focus on individuals’ stimulus appraisals and behavior patterns - which interact to cause and maintain emotional distress and functional impairment (8). Therapeutic strategies aim to (1) flexibilise and expand individuals’ cognitive, emotional and behavioral range, and (2) help individuals to identify and meet their psychological needs through less maladaptive means than psychological symptom expression.

The basic tenets of this therapeutic method have been applied to a broad variety of psychological syndromes. Contrary to what is often assumed, however, “CBTs” are not uniform therapies. Rather, “CBT” is an umbrella term for numerous treatment approaches (9) – which differ in terms of their theories of *etiology* (how does psychopathology emerge?), *process* (how do respective theories of etiology imply ways of change?), and *procedure* (which therapeutic strategies are used to effect change?) (8). CBT approaches include, amongst others, cognitive therapy (CT; 10), problem-solving therapy (11), dialectical behavior therapy (12), metacognitive therapy (13), rational-emotive behavior therapy (14), cognitive processing therapy (15), mindfulness-based cognitive therapy (16), the cognitive-behavioural analysis system of psychotherapy (17), and schema-focused therapy [ST; (18)].

ST adopts a transdiagnostic, emotion-focused perspective, and integrates elements from cognitive, humanistic and psychodynamic treatments in its theory of distress-etiology, process, and change. Unlike “classic” CT, ST emphasizes biographical and relational influences on the development of maladaptive emotional-cognitive-behavioral patterns – so-called “schema modes” (19). These dynamic “self-states” (20) emerge or shift depending on interactions of internal or external circumstances and underlying, more stable personality traits (19). Schema modes can be broadly divided into (1) maladaptive parent, (2) maladaptive child, and (3) maladaptive coping modes as well as (4) a healthy adult mode (21). Maladaptive coping modes can further be grouped into (a) avoidant, (b) subordinating, and (c) overcompensatory strategies. In “parent mode”, individuals’ experiences and behaviours fuse with autobiographically shaped punitive or demanding beliefs; in “child mode” with primary emotions linked to unmet emotional needs; and in “coping mode” with secondary emotions or behaviours which aim to regulate or avoid emotion. Importantly, the schema mode model explicitly postulates that “psychopathology” differs from “normality” merely in terms of frequency and intensity (*not* “presence” or “quality”) of mode activations (18).

In clinical research and practice, the schema mode model is a helpful transdiagnostic concept that allows both patients and therapists to understand (shifts in) their ways of experiencing themselves, others and the world. The therapeutic process is guided by mode-specific goals that involve reducing maladaptive coping modes, facilitating the expression of child modes, and strengthening the healthy adult by means of cognitive, emotion-focused/experiential, and behavioral interventions.

Patients with chronic tinnitus frequently present with psychiatric syndromes – most notably major depressive-, anxiety-, or somatoform disorders (22, 23). Adopting a transdiagnostic perspective these psychiatric “comorbidities” may be understood as differential expressions of underlying, continuously distributed psychological dimensions (24, 25).

In line with ST’s conceptualization as a transdiagnostic approach (26), ST has demonstrated encouraging effects across a variety of syndrome categories – including those commonly encountered in patients with chronic tinnitus (27, 28). Surprisingly, however, STs’ utility has not yet been investigated in this population. Only one paper examined schema modes in patients with chronic tinnitus (29). The study demonstrated high expressions of child, avoidant, and subordinating coping modes, and a conspicuously low expression of the “punitive parent” mode. In extension of these results, the present study investigates (1) underlying dimensions of psychological distress in patients with chronic tinnitus, and (2) the usefulness of the schema mode model to help explain psychological distress variation in this population.

## Methods

### Participants

The present study reports self-report data from *N* = 696 patients who were sampled over a period of two years in routine clinical practice. Note that this paper uses parts of the same data set as reported in (29).

The sample comprised psychological self-report data from adult patients who (a) self-referred to the Tinnitus Centre at Charité Universitätsmedizin Berlin over a two-year period (January 2019 - December 2020), (b) suffered from chronic tinnitus (lasting for > 3 months). Upon joining the center, patients completed a psychological questionnaire battery which included, amongst other measures, German versions of the Tinnitus Questionnaire (TQ), Tinnitus Handicap Inventory (THI), Hospital Anxiety and Depression Scale (HADS), Perceived Stress Questionnaire (PSQ) and ICD-10 Symptom Rating (ISR). Serving as criterion, patients further completed 10 main scales of the revised Schema Mode Inventory (SMI-r). Patients were excluded if they suffered from acute psychotic illness or addiction, (untreated) deafness, or had insufficient knowledge of the German language. Roughly half of the sample (51%) was female. On average, patients were 52 years old (*SD* = 12 years; *range* = 19 – 82 years). Upon arrival at the Tinnitus Centre, patients completed a routine questionnaire assessment battery on electronic tablet devices. Participants provided written consent for data to be collected and used for research purposes, and the Charité Universitätsmedizin Berlin’s ethics committee approved data collection and analysis (No: EA4/216/20). **Table 1** [see also (29)] provides an overview of the sample’s sociodemographic characteristics.

**Table 1:** Sociodemographic information (*N* = 696 patients with chronic tinnitus).

Variable	*M*	*SD*
Age	51.87	12.18
	*n*	*%*
Gender		
Male	338	48.6
Female	358	51.4
Duration of tinnitus		
*<*1/2 year	85	12.2
1/2–1 year	144	20. 7
1–2 years	97	13.9
2–3 years	46	6.6
3–4 years	38	5.5
*>*4 years	286	41.4
Degree		
None	7	1.0
Primary school	9	1.3
General school	42	6.0
O-Levels	118	17.0
A-Levels	60	8.6
Apprenticeship	150	21.6
Polytechnic degree	76	10.9
University degree	234	33.6
Nationality		
German	640	92.0
Other	56	8.0
Relationship status		
Single	164	23.6
In Partnership	70	10.1
Married	341	49.0
Separated	15	2.2
Divorced	89	12.81
Widowed	17	2.4
Work status		
Currently working	493	70.8
Not currently working	203	29.2

M, mean; SD, standard deviation.

### Measures

#### Primary measures

##### Tinnitus

The German version of the Tinnitus Questionnaire (30) is a self-report instrument measuring tinnitus-related distress. The German version consists of 52 items which are rated on a 3-point Likert scale (0 = *not true*, 1 = *partly true*, 2 = *true*). Forty items are used to calculate the total score. Because two items are included twice, the TQ total score ranges from 0 to 84 points. In the current sample, the scale’s internal consistency was excellent (α= 0.94).

##### Tinnitus handicap inventory (THI)

Subjective tinnitus handicap severity was additionally measured by the Tinnitus Handicap Inventory [(31); German version: (32)]. The THI consists of 25 items that are answered on a 3-point scale (0 = *no*; 2 = *sometimes*; 4 = *yes*) resulting in a total score between 0 and 100. In the current sample, the measure’s internal consistency was excellent (α = 0.93).

##### Hospital anxiety and depression scale (HADS)

Anxiety and depressive symptoms were measured using the Hospital Anxiety and Depression Scale (33, 34). The questionnaire combines two 7-item scales that measure anxious or depressive symptoms “during the last week” (0 = “*not at all*” to 3 = “*mostly*”). For the current sample, internal consistencies were good (α_anxiety_ = 0.80; α_depression_ = 0.88).

##### Perceived stress questionnaire (PSQ)

The Perceived Stress Questionnaire (35, 36) assesses subjective stress experiences across four dimensions labelled “tension” [disquietude, exhaustion and lack of relaxation], “worries” [anxious concern for the future, and feelings of desperation and frustration], “joy” [positive feelings of challenge, joy, energy, and security] and “demands” [perceived environmental demands such as lack of time, pressure, and overload]). The scale consists of 30 items that are rated on a 4-point scale (1 = *almost never*, 2 = *sometimes*, 3 = *often*, 4 = *almost always*). All indices are linearly transformed to range from 0 to 100, and averaged into a total score for which *joy* is recoded. In the current sample, internal consistency was excellent (α = 0.94).

##### ICD-10 symptom rating

The ICD-10 Symptom Rating questionnaire measures diagnostic approximations for five psychiatric syndromes as operationalized in the International Classification of Diseases [ICD; (37)]. The ISR comprises 29 items that are answered on a 5-point Likert scale from 0 = strongly disagree to 4 = strongly agree. Item scores are averaged for five subscales that measure depressive, anxiety, obsessive-compulsive, somatoform and eating disorder-related phenomena. Averaging these subscale scores and including an additional scale’s score twice, a total score is computed. For the current sample, the measure’s internal consistency was excellent (α_total_ = 0.92).

### Criterion

#### Schema mode inventory – revised

The revised version of the Schema Mode Inventory (SMI-r) is a 124-item self-report questionnaire which measures the frequency of occurence of 14 schema modes (i.e. cognitive-affective-behavioural self-states) (38). As previously reasoned in (29), we selected the 10 most common modes for reasons of theoretical relevance and in order to reduce response burden. Participants thus rated 86 items on a 6-point Likert scale from 1 = “*never or hardly ever*” to 6 = “*always*”. Ratings were averaged into mode scores – with higher scores indicating higher frequency of mode manifestations.

The present study assessed (1) two maladaptive parent modes: the punitive parent (PP) and the demanding parent (DPT), (2) two maladaptive child modes: the vulnerable child (VC) and the angry child (AC), (3) five maladaptive coping modes that are subdivided into (a) two avoidant coping modes: the detached protector (DP) and the detached self-soother (DSS), (b) one subordinating coping mode: the compliant surrenderer (CS), and (c) two overcompensating modes: the self-aggrandizer (SA), and bully-and-attack (BA). Last, healthy emotion-regulation abilities and cognitive-affective resources were measured via (4) the healthy adult (HA).

On an individual’s inner stage, the PP devalues, criticizes, blackmails, and punishes the self or others. The DPT places high responsibilities and standards on the self or others and pressures the individual never to make mistakes or fail to live up to (others’) high expectations.

The VC experiences deep feelings of loneliness, fear, unhappiness, and helplessness; whilst the AC feels infuriated, angry, and indignant that the VC’s needs are not met.

The DP avoids, and withdraws or disconnects from both emotional states and meaningful human contact. The DP can involve ‘cognitive’ ways of dealing with emotional distress (e.g. being overly rational or intellectualizing, superficial, vague or in demand of concrete ‘solutions’). The DSS has a similar agenda, yet uses repetitive, seemingly pleasurable activities to distance a person from emotions or meaningful connections.

The CS appears to comply with others’ demands in order to avoid emotional vulnerability. Whilst appearing subordinate, passive, or dependent, the CS may equally be characterized by strong indirect expressions of anger and self-assertion.

The SA feels superior, special, and powerful. It stabilizes the self by dismissing or devaluing others’ opinions, feelings, or needs; and may resort to boastful or exploitative behaviors to achieve a sense of safety. The BA Mode aims to secure the vulnerable child by humiliating, intimidating, and destroy other people – both psychologically and behaviorally.

The German version of the SMI-r yields good-to-excellent internal consistency and construct validity (39). In the current sample, internal consistencies were acceptable-to-excellent (α_punitive parent_ = 0.84; α_demanding parent_ = 0.84; α_vulnerable child_ = 0.93; α_angry child_ = 0.84; α_detached protector_ = 0.86; α_detached self-soother_ = 0.72; α_compliant surrenderer_ = 0.73; α_self-aggrandizer_ = 0.79; α_bully and attack_ = 0.79; α_healthy adult_ = 0.81).

### Data analysis

Analyses were conducted using IBM SPSS Statistics for Windows, Version 24. First, we report descriptive statistics and correlation coefficients *r* for the primary measures. Note that the reported descriptives in **Tables 2** and **3** were previously reported in (29) - and are repeated here to provide background for the interpretation of the factor analysis. Next, the overall item-pool of the TQ, THI, PSQ, HADS, and ISR (128 items) underwent a principal axis factor analysis with *varimax* rotation. Following the identification of an appropriate factor solution, we computed factor scores as the mean of the respectively loading items per factor^1^. These factor scores were then correlated with the SMI-r to aid factor interpretation by means of the schema mode model. Correlation coefficients were defined as negligible (0.00–0.29), low (0.30 – 0.49), moderate (0.50–0.69), high (0.70–0.89) or very high [≥ 0.90; (40)].

**Table 2:** Means and standard deviations for the examined questionnaires for *N* = 696 patients with chronic tinnitus.

	*M*	*SD*	Clinical classifications (*M* ± 1 *SD*)
TQ	40.04	16.32	mild-moderate
THI	45.35	22.17	moderate-severe
HADS_a	8.53	4.14	mild-moderate
HADS_d	7.07	4.68	mild-moderate
PSQ	50.97	20.19	mild-moderate
worries	43.05	24.15	normal-moderate
tension	59.29	23.53	normal-moderate
joy	46.51	23.61	normal-moderate
demands	48.07	24.70	normal-mild
ISR	1.00	0.59	normal-moderate
depression	1.48	0.95	normal-moderate
anxiety	1.25	0.95	normal-moderate
obsessive- compulsive	0.93	0.88	normal-mild
somatoform	0.83	0.88	normal-moderate
eating- related	0.69	0.81	normal-moderate
SMI-r			
punitive parent	1.71	0.63	highly decreased^*^
demanding parent	3.00	1.03	mildly increased^*^
vulnerable child	2.16	0.95	moderately increased^*^
angry child	2.27	0.79	moderately increased^*^
detached protector	2.15	0.77	moderately increased^*^
detached self-soother	2.77	0.99	moderately increased^*^
compliant surrenderer	2.77	0.73	moderately increased^*^
self- aggrandizer	2.28	0.67	normal^*^
bully-attack	1.65	0.57	mildly increased^*^
healthy adult	4.26	0.75	moderately decreased^*^

* = relative to a non-clinical German reference sample [(39); see also (29)].

M, mean; SD, standard deviation; PP, punitive parent mode; DBT, demanding parent mode; HADS_a, Hospital Anxiety and Depression Scale-Anxiety subscale; HADS_d, Depression subscale; ISR, ICD-10 Symptom Rating; PSQ, Perceived Stress Questionnaire; THI, Tinnitus Handicap Inventory; TQ, Tinnitus Questionnaire (German version); SMI-r, Schema Mode Inventory-revised.

**Table 3:** Pearson correlation coefficients for the primary psychological measures (all *p* <.001).

	THI	HADS_a	HADS_d	PSQ					ISR					
					worries	tension	joy	demands		depression	anxiety	obsessive-compulsive	somatoform	eating-related
TQ	0.88	0.57	0.59	0.47	0.47	0.47	-0.41	0.23	0.60	0.55	0.46	0.34	0.43	0.22
THI		0.63	0.66	0.59	0.59	0.55	-0.53	0.31	0.68	0.65	0.52	0.43	0.50	0.20
HADS_a			0.69	0.73	0.73	0.70	-0.63	0.41	0.76	0.70	0.67	0.54	0.50	0.22
HADS_d				0.70	0.69	0.61	-0.77	0.29	0.72	0.81	0.51	0.47	0.40	0.18
PSQ					0.88	0.89	-0.84	0.76	0.72	0.76	0.53	0.50	0.41	0.22
worries						0.71	-0.71	0.54	0.75	0.75	0.56	0.50	0.46	0.23
tension							-0.71	0.59	0.65	0.69	0.47	0.45	0.36	0.20
joy								-0.42	-0.67	-0.75	-0.49	-0.47	-0.34	-0.16
demands									0.38	0.38	0.27	0.28	0.22	0.15
ISR										0.82	0.79	0.74	0.69	0.44
depression											0.59	0.53	0.44	0.19
anxiety												0.54	0.49	0.19
obsessive- compulsive													0.41	0.21
somatoform														0.22

HADS_a, Hospital Anxiety and Depression Scale-Anxiety subscale; HADS_d, Depression subscale; ISR, ICD-10 Symptom Rating; PSQ, Perceived Stress Questionnaire; THI, Tinnitus Handicap Inventory; TQ, Tinnitus Questionnaire (German version). Principal axis factor analysis.

## Results

### Descriptive indices

**Table 2** reports means and standard deviations for the completed questionnaires. Overall, the questionnaire scores yielded relatively broad distributions suggesting substantial psychological heterogeneity (41).

**Table 3** reports Pearson’s correlation coefficients (*r*) between the primary outcome measures. All measures correlated at *p* <.001.

**Figure 1:**
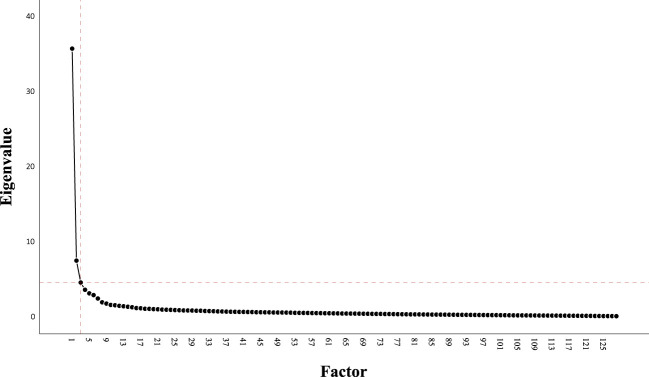
Scree plot of principal axis factor analysis with varimax rotation for N = 696 patients with chronic tinnitus on all items from the TQ, THI, HADS_a, HADS_d, PSQ, and ISR.

To identify latent psychological dimensions, a *varimax*-rotated principal axis factor analysis was conducted on the 128 items of the primary measures (TQ, THI, HADS_a, HADS_d, PSQ, and ISR). The Kaiser-Meyer-Olin measure verified the sampling adequacy for the analysis [*KMO* = 0.96; ‘*marvelous’* (42)]. The scree plot was ambiguous and showed inflexions that would justify a 3- or 4-factor solution (see **Figure 1**). Because of the relatively small sample size and small relative increase in explained variance, we opted for the 3-factor solution (37.41% of the variance [vs. 40.25% for the 4-factor solution^2^]).

Following item-content screening, Factor 1 likely represents “**General emotional distress**” (27.93% of variance; highest loading items: “*I feel happy*”, “*You have fun*” [-0.78, -0.76]), Factor 2 “**Tinnitus-attributed emotional distress**” (5.88% of variance; highest loading items: “*Are you depressed because of the tinnitus*?”, “*Do you feel desperate because of the tinnitus*?” [0.69, 0.68]), and Factor 3 “**Socio-audiological impairment**” (3.60% of variance; highest loading items: “*Because of the tinnitus, it is more difficult for me to follow a conversation*”, “*Does the volume of your tinnitus prevent you from understanding other people*?” [0.81, 0.79]). For an overview of items and factor loadings, see Online **Supplementary 1**.

**Figure 2:**
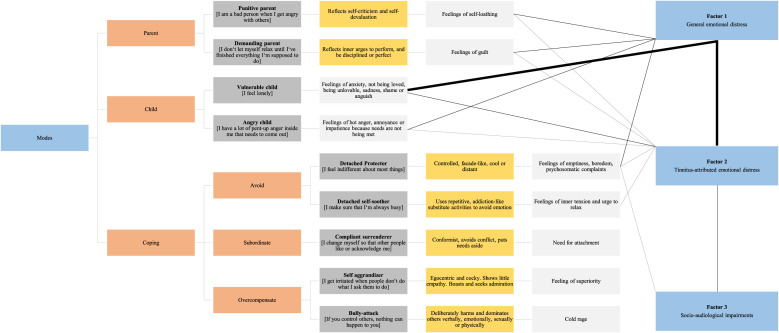
The schema mode model (left) and identified dimensions of psychological distress (right). Orange boxes indicate superordinate mode groups, dark grey boxes associated modes and exemplary measurement items, yellow boxes the “quality” of the mode as reflected in the actualized clinical patient presentation, and light grey boxes associated emotions or emotional needs respectively. Strong black lines represent high (0.70 < *r* < 0.90), thin black lines moderate (0.50 < *r* < 0.69), and grey lines low correlations [0.30 < *r* < 0.49; (40)].

To examine, if the factor solution could be interpreted within the schema mode model, correlation coefficients *r* examined associations between the factor scores and the SMI-r (see **Table 4** and **Figure 2**). *High correlations* emerged between Factors 1 and 2, as well as Factor 1 and the "vulnerable child". *Moderate correlations* emerged for Factors 2 and 3; Factor 1 and the “parent”, “angry child”, and “detached protector”; and Factor 2 and the “vulnerable child”. Factor 3 was largely uncorrelated with SMI-r scores – although a low correlation with the “detached protector” warrants further examination.

**Table 4:** Pearson correlation coefficients for the factor scores and the SMI-r.

Factor		2	3	PP	DPT	VC	AC	DP	DSS	CS	SA	BA	HA
**Factor 1**	*General emotional distress*	0.70	0.48	0.60	0.53	0.78	0.58	0.65	0.44	0.28	0.46	0.29	-0.48
**Factor 2**	*Tinnitus-attributed emotional distress*	–	0.62	0.43	0.35	0.53	0.41	0.41	0.37	0.20	0.28	0.24	-0.39
**Factor 3**	*Socio-audiological impairment*	–	–	0.22	0.20	0.28	0.18	0.31	0.18	-	0.25	0.08^*^	-0.26

Coefficients are significant at *p* <.001 unless otherwise indicated. ^*^ = *p* <.05; Red coefficients emphasize high (0.70 < *r* < 0.90), orange coefficients moderate (0.50 < *r* < 0.69); and light grey low correlations [0.30 < *r* < 0.49; (40)]. PP, punitive parent mode; DBT, demanding parent mode; VC, vulnerable child mode; AC, angry child mode; DP, detached protector mode; DSS, detached self-soother mode; CS, compliant surrenderer mode; BA, bully-attack mode; HA, healthy adult mode; SMI-r, Schema Mode Inventory (revised).

## Discussion

The present study examined self-report data from *N* = 696 patients with chronic tinnitus. Participants completed measures of tinnitus-related distress, anxiety, depression, perceived stress, and psychological symptoms across a variety of common psychiatric syndrome diagnoses. The study aimed to identify transdiagnostic dimensions of psychological distress – and whether schema modes might help formulate these dimensions.

In keeping with common observations in this patient population (41), all psychological distress measures (a) intercorrelated highly and (b) yielded broad confidence intervals suggesting high levels of heterogeneity. Factor-analytic models exploit such correlational patterns in order to empirically identify psychological, behavioral or physiological risk factors for psychopathology (43, 44). The present study used a *varimax*-rotated principal axis factor analysis and identified a three-factor structure which explained 37.41% of variance. The factors likely represented (1) **General emotional distress**, (2) **Tinnitus-attributed emotional distress**, and (3) **Socio-audiological impairment**. Factors 1|2 correlated highly at *r* = 0.70, Factors 2|3 moderately at *r* = 0.62. Conceptually, the measures’ indices of “anxiety”, “depression”, “perceived stress”, and “psychiatric syndromes” all constitute differential expressions of a common underlying factor – which may either precede or ‘incorporate’ the tinnitus symptom (45, 46). The current factor solution somewhat reflects results from a previous study that labelled “stress”, “pain experience”, “fatigue”, “autonomy”, and “educational level” as dimensions of tinnitus-related distress in a sample of *N* = 1958 patients with chronic tinnitus (47).

Clinically, the data support the importance of formulating the tinnitus symptom in *context* of individuals’ autobiographical and current distress experiences (48–50). Consequently, psychologists and psychotherapists ought to move beyond somewhat simplistic symptom-specific (“depressed because of tinnitus”) or categorical, syndrome-specific understandings of emotional distress (“treat ‘the tinnitus’ – then ‘the depression’”). Rather, it is important to adopt a person- (not symptom-) centered perspective that (1) shifts its therapeutic focus away from the tinnitus symptom towards its idiographic appraisal and assigned meaning in context of individuals’ biographies and current lives, and (2) adopts an emotion-focused stance in order to understand and ameliorate patients’ distress in context of their autobiographical, affective and relational complexity (51–53).

One such perspective is offered by so-called “schema mode” approach – which is located in the overall landscape of cognitive-behavioural therapies (18, 21, 54). In the present study, the “vulnerable child” accounted for substantial proportions of psychological distress (61% of Factor 1 and 28% of Factor 2’s variance). Thus, patients’ distress experiences appear to be characterized by feelings of inadequacy, worthlessness, despair, loneliness, fear, and helplessness. These emotional states (1) may reflect difficult biographical experiences (as indexed by moderate correlations between Factor 1 and the parent modes) and (2) are primarily managed by the “detached protector” (as similarly indexed by its moderate correlation with Factor 1). Whilst the importance of these feelings has been previously highlighted (55), the available literature tends to link (and discuss) these feelings exclusively (with regard) to the tinnitus-symptom. In doing so, correlation and causation are frequently confounded at the expense of (1) biographical experiences that shape the habitual appraisal of stimuli as well as unmet emotional needs (which inform symptom function), and (2) third variables that may influence both tinnitus-symptom onset and tinnitus-distress variability (56–58). Overall, therapeutic endeavors ought to (1) focus on reducing emotional avoidance and (2) explore the symptom’s intrapsychic and interpersonal functions en route to discovering and integrating distressing affective states into the healthy adult mode.

The moderate correlation between Factors 2|3 suggests that “socio-audiological impairment” (Factor 3) interacts with tinnitus-attributed distress - and thus represents an important psychosocial treatment goal. Although hearing-related difficulties undoubtedly underlie a major part of audiological impairments and initial tinnitus-symptom onset (3), psychological factors also influence both groups of phenomena (59–63). Whilst current treatment guidelines recommend hearing amplification measures for patients with chronic tinnitus and hearing loss (5, 6), it is crucial to conceptualize hearing loss with a view to its broader socio-psychological consequences (60, 62, 64, 65). In our data, the small-yet-significant correlation between Factor 3 and the “detached protector” raises the interesting possibility that emotional avoidance may interact with socio-audiological difficulties – which thus ought to be conceptualized holistically.

The current study has several limitations. For example, self-report data feature common limitations [(66); yet see also 67)]. In addition, factor analytic results and factor interpretations are never “true”. Rather, they depend on the selection and content of the originally included item set, as well as statistical and theoretical considerations regarding the number and nature of the extracted factors. Last, factor analyses are a regression-based method which disallows causal conclusions. Thus, future studies need to demonstrate the clinical usefulness of schema therapeutic approaches in addressing common underlying factors of emotional distress in patients with chronic tinnitus. Clinically and crucially, a mechanistic or medicalized view of psychological interventions risks underestimating or ignoring strong evidence for relational humanistic factors that actually make psychotherapy effective (68–71).

Nonetheless, the present study (1) replicates and expands previous factor analytic work regarding tinnitus-related psychopathology (47), (2) identifies three transdiagnostic psychological dimensions, (3) links these constructs to a potentially useful emotion-focused “new” cognitive-behavioural therapy framework (54, 72), and, thereby (4) encourages future empirical examination of short-term group or individual schema-therapy-based interventions in patients with chronic tinnitus (73–75). With its dynamic focus on intra- and interpersonal cognitive-emotional states, the schema-mode model offers a useful approach for researchers and clinicians alike to conceptualize and alleviate emotional distress in patients with chronic tinnitus.

## Data availability statement

The datasets presented in this article are not readily available. As per Charité Universitaetsmedizin Berlin’s ethics committee, the data cannot be made public without restrictions because patients did not give consent at the time. Requests to access the data should be directed to the directorate of the Tinnitus Center Charité Universitaetsmedizin Berlin (birgit.mazurek@charite.de) or Charité's Open Data and Research Data Management Officer, Dr. Evgeny Bobrov (evgeny.bobrov@charite.de).

## Ethics statement

The studies involving humans were conducted in accordance with the Declaration of Helsinki, and approved by the ethics committee of Charité Universitatsmedizin Berlin (EA4/216/20). The studies were conducted in accordance with the local legislation and institutional requirements. The participants provided their written informed consent to participate in this study.

## Author contributions

BB: Writing – review & editing, Writing – original draft, Visualization, Project administration, Methodology, Formal analysis, Data curation, Conceptualization. PB: Writing – review & editing. BM: Writing – review & editing, Supervision, Resources.

## Footnotes

1 Items who loaded on more than one factor, were included once in the factor score of their highest loading.

2 Factor 4 appeared to reflect "Overstraining" (5 items [PSQ 3, 20, 2, 19, 11]; 2.84% of variance; highest loading items “*You have too much to do*”, “*You feel under deadline pressure*” [0.80, 0.77]) – and correlated moderately with Factor 1 (*r* = 0.51) and the “demanding parent” mode (*r* = 0.54).
