# Supplementary material for: “I feel [so alone] nothing” – emotional vulnerability and detachment as transdiagnostic key characteristics of patients with chronic tinnitus: a schema mode model approach
Source: Front Psychiatry. 2024 Jun 18;15:1375813. doi: 10.3389/fpsyt.2024.1375813 (PMC11229517; doi:10.3389/fpsyt.2024.1375813)
Supplement: Supplementary file 1 [file Table_1.docx]

**Online Supplement 1.** Summary of varimax-rotated principal axis factor analysis (*N* = 696 patients with chronic tinnitus) across items from the TQ, THI, HADS_a, HADS_d, PSQ and ISR.

| **Questionnaire** | **Original item no** | **Items** (retranslated [*not* original wording]) | **Rotated Factor Loadings** | | |
| --- | --- | --- | --- | --- | --- |
|  |  |  | **1** | 2 | 3 |
| HADS | 6 | I feel happy | -0.78 |  |  |
| PSQ | 14 | You have fun | -0.76 |  |  |
| HADS | 12 | I look to the future with joy | -0.72 |  |  |
| ISR | 2 | I no longer take real pleasure in things I normally like to do | 0.71 |  |  |
| ISR | 1 | My mood is depressed / dejected | 0.70 | 0.32 |  |
| HADS | 2 | I can still be as happy today as I used to be | -0.68 |  |  |
| PSQ | 16 | You have a light heart | -0.66 |  |  |
| PSQ | 12 | You feel safe and protected | 0.66 |  |  |
| PSQ | 8 | You are full of energy | -0.65 |  |  |
| HADS | 4 | I can laugh and see the funny side of things | -0.64 |  |  |
| PSQ | 7 | You feel frustrated | 0.63 |  |  |
| ISR | 4 | I feel worthless/not confident in myself | 0.62 |  |  |
| PSQ | 4 | You feel like doing things you really like | -0.60 |  |  |
| ISR | 3 | I don't have enough energy when I want to do something and get tired easily | 0.60 |  |  |
| PSQ | 15 | You are afraid of the future | 0.60 | 0.30 |  |
| HADS | 8 | I feel slowed down in my activities | 0.58 |  |  |
| PSQ | 17 | You feel mentally exhausted | 0.57 |  |  |
| HADS | 5 | I have worrying thoughts running through my head | 0.55 |  |  |
| PSQ | 1 | You feel rested | -0.55 |  |  |
| PSQ | 13 | You have many worries | 0.54 |  |  |
| PSQ | 10 | Your problems seem to pile up | 0.53 |  |  |
| HADS | 14 | I can enjoy a good book, radio or television programme | -0.52 |  |  |
| HADS | 1 | I feel tense or overstimulated | 0.52 |  |  |
| HADS | 7 | I can sit comfortably and relax | -0.52 |  |  |
| PSQ | 6 | I feel calm | -0.51 |  |  |
| ISR | 24 | I have psychological problems due to heavy everyday stresses (e,g, serious illness, loss of job or separation from partner) | 0.49 |  |  |
| ISR | 18 | I have difficulties concentrating | 0.48 |  |  |
| PSQ | 18 | You have problems relaxing | 0.47 |  |  |
| PSQ | 9 | You feel tense | 0.47 |  |  |
| PSQ | 5 | You fear not being able to achieve your goals | 0.47 |  |  |
| ISR | 27 | I feel impaired in the exercise of my sexuality | 0.46 |  |  |
| ISR | 11 | I suffer from distressing, senseless thoughts or actions that interfere with my normal life | 0.43 |  |  |
| ISR | 22 | I am forgetful | 0.39 |  |  |
| HADS | 3 | I am overcome by a fearful premonition that something terrible might happen | 0.38 |  |  |
| HADS | 10 | I have lost interest in my physical appearance | 0.37 |  |  |
| HADS | 9 | I sometimes have an anxious feeling in my stomach area | 0.37 |  |  |
| ISR | 9 | I suffer from repetitive, senseless thoughts or actions that I can't help myself from (e.g. washing my hands) | 0.35 |  |  |
| HADS | 13 | I am suddenly overcome by a panic-like state | 0.35 |  |  |
| ISR | 5 | I get unexplained anxiety attacks or fear in situations that seem harmless to other people | 0.35 |  |  |
| ISR | 23 | I suffer from recurring dreams or memories of horrible experiences | 0.34 |  |  |
| ISR | 21 | I have a poor appetite | 0.33 |  |  |
| ISR | 13 | I suffer from the constant nagging worry of being physically ill | 0.32 | 0.31 |  |
| PSQ | 2 | You have the feeling that too many demands are being made on you | 0.32 |  |  |
| PSQ | 11 | You feel rushed | 0.32 |  |  |
| ISR | 19 | I think about killing myself | 0.31 |  |  |
| ISR | 8 | The very idea that I could have another anxiety attack scares me | 0.31 |  |  |
|  |  |  | 1 | **2** | 3 |
| THI | 21 | Are you depressed because of the tinnitus? | 0.34 | 0.69 |  |
| THI | 5 | Do you feel desperate because of the tinnitus? |  | 0.68 |  |
| THI | 10 | Do you feel frustrated because of the tinnitus? | 0.34 | 0.67 |  |
| TQ | 47 | I am a victim of my tinnitus |  | 0.66 |  |
| TQ | 39 | I am more easily depressed because of the tinnitus |  | 0.63 |  |
| THI | 3 | Does the tinnitus make you angry? |  | 0.63 |  |
| TQ | 41 | Because of the tinnitus, life seems to be getting on top of me | 0.30 | 0.63 |  |
| THI | 23 | Do you feel that you can no longer cope with the tinnitus? |  | 0.62 |  |
| THI | 22 | Does the tinnitus frighten you? |  | 0.62 |  |
| TQ | 13 | I worry if I will ever be able to cope with this problem |  | 0.62 |  |
| TQ | 37 | When I think about the tinnitus, I sometimes get very angry |  | 0.60 |  |
| TQ | 43 | I often think about whether the tinnitus will ever go away |  | 0.59 |  |
| TQ | 8 | I worry that the tinnitus will drive me into a nervous breakdown |  | 0.58 |  |
| TQ | 27 | It will be terrible if the ringing never goes away |  | 0.58 |  |
| TQ | 17 | If the tinnitus continues, my life will no longer be worth living |  | 0.56 |  |
| TQ | 28 | I worry that the tinnitus will damage my physical health |  | 0.55 |  |
| THI | 16 | Does the tinnitus upset you? |  | 0.54 |  |
| THI | 11 | Does the tinnitus makes you feel like you are suffering from a terrible disorder? |  | 0.54 |  |
| THI | 8 | Do you feel that you cannot escape the tinnitus? |  | 0.53 |  |
| THI | 12 | Does the tinnitus make it difficult for you to enjoy your life? | 0.39 | 0.52 |  |
| THI | 6 | Do you complain a lot about the tinnitus? |  | 0.51 |  |
| TQ | 3 | It is unfair that I have to suffer from my tinnitus |  | 0.50 |  |
| TQ | 18 | I have lost some of my self-confidence because of the tinnitus |  | 0.49 |  |
| THI | 25 | Does the tinnitus make you feel insecure? |  | 0.48 | 0.34 |
| THI | 14 | Do you have the impression that you are often irritable because of the tinnitus? |  | 0.44 |  |
| THI | 18 | Do you find it difficult to focus your attention on other things than your tinnitus? |  | 0.43 |  |
| THI | 17 | Do you have the impression that your tinnitus problem is also putting a strain on your relationship with your family and friends? |  | 0.42 | 0.36 |
| TQ | 21 | There is very little you can do to cope with the tinnitus |  | 0.42 |  |
| TQ | 19 | I wish someone would understand what the problem is |  | 0.41 |  |
| TQ | 34 | I find it harder to relax because of the tinnitus |  | 0.40 |  |
| TQ | 16 | I worry if there is something seriously wrong with my body because of the tinnitus |  | 0.40 |  |
| TQ | 35 | Often my tinnitus is so bad that I can't ignore it |  | 0.38 |  |
| TQ | 11 | I have the impression that I can never escape the tinnitus |  | 0.38 |  |
| THI | 4 | Does the tinnitus confuse you? |  | 0.38 |  |
| TQ | 20 | No matter what I do, the tinnitus distracts me |  | 0.37 | 0.32 |
| TQ | 44 | I can imagine learning to cope with the tinnitus |  | 0.36 |  |
| TQ | 48 | The tinnitus has affected my concentration |  | 0.35 | 0.32 |
| THI | 20 | Are you tired because of the tinnitus? |  | 0.35 |  |
| THI | 15 | Do you find it difficult to read because of the tinnitus? |  | 0.33 |  |
| THI | 19 | Do you feel that you have no control over the tinnitus? |  | 0.32 |  |
| TQ | 10 | The way the tinnitus sounds is really unpleasant |  | 0.31 |  |
| TQ | 36 | It takes me longer to fall asleep because of the tinnitus |  | 0.30 |  |
|  |  |  | 1 | 2 | **3** |
| TQ | 33 | Because of the tinnitus, it is more difficult for me to follow a conversation |  |  | 0.81 |
| THI | 2 | Does the volume of your tinnitus prevent you from understanding other people? |  |  | 0.79 |
| TQ | 14 | Because of the tinnitus, it is more difficult for me to listen to several people at the same time |  |  | 0.77 |
| TQ | 9 | Because of the tinnitus, I have difficulty telling where other sounds are coming from |  |  | 0.64 |
| THI | 9 | Does the tinnitus interfere with your social life (e.g., going out for dinner, going to the cinema)? |  |  | 0.62 |
| TQ | 38 | I find it harder to talk on the phone because of the tinnitus |  |  | 0.61 |
| TQ | 26 | Other people's voices seem distorted to me because of the tinnitus |  |  | 0.54 |
| TQ | 50 | I am unable to enjoy the radio or television because of the tinnitus |  |  | 0.51 |
| TQ | 2 | I cannot enjoy music because of the tinnitus |  |  | 0.47 |
| THI | 13 | Does the tinnitus prevent you from doing your work or household tasks? |  | 0.38 | 0.41 |
| THI | 1 | Do you find it difficult to concentrate because of the tinnitus? |  | 0.33 | 0.35 |
| TQ | 15 | The tinnitus is loud most of the time |  |  | 0.34 |

*Notes*. HADS = Hospital Anxiety and Depression Scale; ISR = ICD-10 Symptom Rating; PSQ = Perceived Stress Questionnaire; THI = Tinnitus Handicap Inventory; TQ = Tinnitus Questionnaire
